# Genetic evidence of illegal trade in protected whales links Japan with the US and South Korea

**DOI:** 10.1098/rsbl.2010.0239

**Published:** 2010-04-14

**Authors:** C. Scott Baker, Debbie Steel, Yeyong Choi, Hang Lee, Kyung Seok Kim, Sung Kyoung Choi, Yong-Un Ma, Charles Hambleton, Louie Psihoyos, R. L. Brownell, Naoko Funahashi

**Affiliations:** 1 Marine Mammal Institute and Department of Fisheries and Wildlife, Oregon State University, Newport, OR 97365, USA; 2 Ocean Committee, Korean Federation for Environmental Movements, Seoul, South Korea; 3 Conservation and Genome Resource Bank for Korean Wildlife, Seoul National University, Seoul, South Korea; 4 Nature Conservation Committee, Korean Federation for Environmental Movements, Seoul, South Korea; 5 Oceanic Preservation Society, Boulder, CO, USA; 6 National Marine Fisheries Service, Southwest Fisheries Science Center, Pacific Grove, CA, USA; 7 International Fund for Animal Welfare, Tokyo, Japan

**Keywords:** IWC, whaling, CITES, infraction

## Abstract

We report on genetic identification of ‘whale meat’ purchased in sushi restaurants in Los Angeles, CA (USA) in October 2009 and in Seoul, South Korea in June and September 2009. Phylogenetic analyses of mtDNA cytochrome *b* sequences confirmed that the products included three species of whale currently killed in the controversial scientific whaling programme of Japan, but which are protected from international trade: the fin, sei and Antarctic minke. The DNA profile of the fin whale sold in Seoul established a match to products purchased previously in Japan in September 2007, confirming unauthorized trade between these two countries. Following species identification, these products were handed over to the appropriate national or local authorities for further investigation. The illegal trade of products from protected species of whales, presumably taken under a national permit for scientific research, is a timely reminder of the need for independent, transparent and robust monitoring of any future whaling.

## 1. Introduction

The hunting of large whales is regulated by the International Whaling Commission (IWC), and trade in whale products is regulated by the Convention on International Trade in Endangered Species (CITES). In 1982, the IWC adopted a moratorium on commercial whaling, effective from 1986. However, hunting of whales continues through exceptions to the moratorium. Japan initiated hunting of Antarctic minke whales under Special Permit (i.e. ‘scientific whaling’) in 1988, and has steadily expanded this programme to include western North Pacific common minke whales, sei whales, Bryde's whales, sperm whales and, most recently, fin whales from the Antarctic (see the electronic supplementary material S1, table S1). Japan and the Republic of Korea (South Korea) allow a thriving commercial sale of whales killed in incidental fisheries ‘bycatch’ (Lukoschek *et al*. 2009). Iceland and Norway initiated whaling under Special Permit following the moratorium but now conduct commercial whaling in the North Atlantic under an ‘objection’ to the moratorium.

Although whaling and the domestic sale of whale products continue despite the IWC moratorium, it is generally assumed that there is no ongoing international trade in whale products. All 13 species of whales regulated by the IWC are listed on appendix I of CITES, except for the West Greenland population of minke whale. Appendix I species cannot be traded for commercial purposes. However, Japan, Iceland and Norway maintain ‘reservations’ on the appendix I listing of some whales, allowing trade of products from these species with ‘appropriate’ permits. These exceptions do not allow trade with countries that do not hold such CITES reservation, such as South Korea and the US.

Given the number of ongoing and emerging exceptions to the hunting of whales and to the trade in whale products, there is an urgent need for effective measures to verify authorized catch limits and trade records, and to detect infractions. Among the most powerful tools for the control of trade in fisheries and wildlife are molecular monitoring of commercial outlets (e.g. markets and restaurants) and genetic tracking of products (Baker 2008). Through sequencing of mitochondrial (mt) DNA, or ‘DNA barcoding’, it is possible to identify the species origin of almost any cetacean product. Through multi-locus genotyping, or ‘DNA profiling’, it is possible to identify the individual source of a product or to track the distribution of products from source to market (Cipriano & Palumbi 1999; Baker *et al*. 2007).

A formal extension of genetic tracking involves establishing a ‘DNA register’, or electronic database, to include the DNA profile of all individual whales destined for trade or commercial sale (DeSalle & Amato 2004). The DNA profile of a questionable market product or customs interdiction can then be compared with the register; a match to the register would confirm that the product originated from an authorized source and was imported with appropriate permits. Japan and Norway have both developed DNA registers for whales destined for commercial sale. However, both countries oppose implementation of market surveys for monitoring of whaling and neither country has yet agreed to the sharing of the register with an independent central authority (e.g. the IWC Secretariat). Requests for access to the registers are by application to the appropriate national authorities.

Here, we report on genetic evidence linking ‘whale meat’ products purchased in sushi restaurants in Los Angeles, USA and in Seoul, South Korea, to a probable origin in the scientific whaling programme of Japan. The purchases of the products from these two restaurants were opportunistic and the timing was coincidental, as each involved investigators who were unknown to each other. To allow genetic tracking of these products, we have submitted a request to the Government of Japan for access to the DNA register of whales taken in scientific whaling and in commercial ‘bycatch’ whaling.

## 2. Material and methods

Products advertised as originating from whale were purchased in Japanese restaurants in Seoul, South Korea and the greater Los Angeles (LA) area (see the electronic supplementary material S1, table S2). Products from the Seoul restaurant were stored in 70 per cent EtOH for analysis on location in South Korea and subsequent transfer to the Conservation Genome Resource Bank at Seoul National University. Products from the LA restaurant were shipped frozen to the Laboratory of Conservation Genetics at Oregon State University.

DNA extraction and amplification followed the ‘portable PCR’ protocols used previously in market surveys (Baker *et al*. 2006). We used both mtDNA cytochrome *b* sequences for species identification, and control region sequences and seven published microsatellite loci for individual DNA profiles (see the electronic supplementary material S1, table S3). The species origin of each product was identified using the web-based programme, DNA Surveillance (Ross *et al*. 2003), and confirmed by BLAST search of the international genetic archive, GenBank. Matching of DNA profiles and calculation of the probability of identity were conducted with the programme Cervus (Marshall *et al*. 1998). The cytochrome *b* sequences used for species identification have been deposited with GenBank as numbers HM034289–HM034303 (see the electronic supplementary material S2). DNA profiles have been embargoed, pending proposal for matching to DNA registers (see the electronic supplementary material S3).

## 3. Results and discussion

### (a) Los Angeles, USA

**Figure 1.**
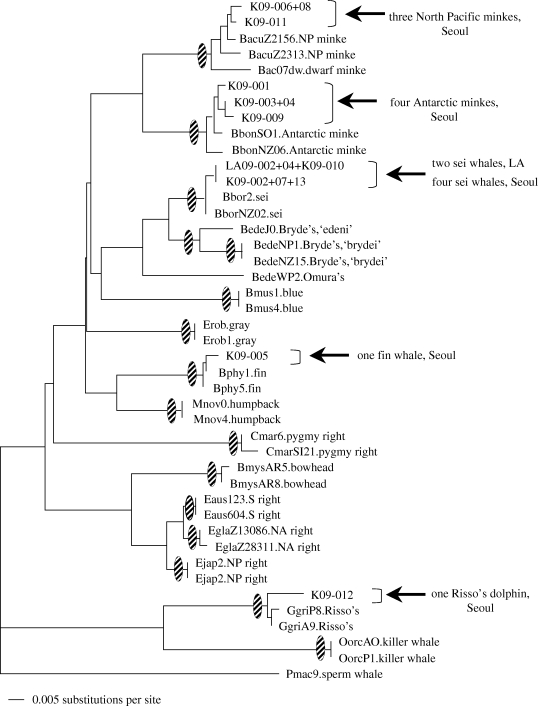
Phylogenetic identification of ‘whalemeat’ products based on mtDNA cytochrome *b* gene (approximately 400 base pairs in length, see the electronic supplementary material S2), as implemented in www.DNA-surveillance (database, v. 4.3). Shaded ovals indicate species-specific groupings supported by more than 90% of 1000 bootstrap simulations in a neighbour-joining reconstruction.

Four strips of raw meat (sashimi) were purchased by one of the authors (CH) at a renowned sushi restaurant in the Los Angeles area in October 2009. The products were offered only to those willing to be ‘adventurous’ and were hand-written on the sales receipt as originating from ‘whale’ and ‘horse’. Species identification for both control region and cytochrome *b* sequences was consistent and conclusive (figure 1): the two pieces of raw meat offered as whale were identified as sei whale; and, the two pieces offered as horse were identified as a domestic cow.

Given the limited number of reference sequences for sei whales available in both GenBank and DNA-Surveillance, we also compared the sequences from the LA restaurant with those from products purchased during molecular monitoring of Japanese whalemeat markets (see the electronic supplementary material S1, table S2). The comparison showed that the sequences were identical to products purchased in Japan in 2007 and 2008, consistent with an origin from a whale killed in the JARPNII hunt (Japanese scientific hunt) of sei whales in the North Pacific. Following the initial species identification, the products were turned over to national authorities (National Oceanic and Atmospheric Administration, Office of Law Enforcement) for further investigation. On 10 March 2010, federal prosecutors filed a criminal complaint against the restaurant and its chef.

### (b) Seoul, South Korea

A total of 13 whalemeat products were purchased at a sushi restaurant in the central district of Seoul during two visits: the first in June 2009 (by YC), and the second in September (by NF, KSK, HL and CSB). The restaurant menu included an extensive offering of whalemeat specialty dishes, including a ‘mixed plate’ of sashimi whalemeat. Species identification for both control region and cytochrome *b* sequences was consistent and conclusive: four products were Antarctic minke whale, four were sei whale, three were North Pacific minke whale, one was fin whale and one was Risso's dolphin (figure 1).

Unlike most previous surveys of Korean whalemeat markets in Busan, Ulsan and Pohang (e.g. Baker *et al*. 2006), some of the species sold in the Seoul restaurant are inconsistent with a local origin. The Antarctic minke whale is not found in waters of the Northern Hemisphere. The sei whale has not been found in previous surveys of Korean markets or reported in the 13 years of official record of bycatch submitted by the Government of Korea to the IWC. The fin whale has not been found previously in surveys of Korean markets and only twice as bycatch in Korea records, once in 2002 and once in 2004. Following initial species identification, the products were turned over to local authorities (Seoul Metropolitan Police) for further investigation.

### (c) Links with Japan

We investigated a direct link in illegal trade between Korea and Japan by comparing the DNA profiles of product purchased in the Seoul restaurant to those of fin whale products purchased during surveys of the Japanese market from September 2006 to April 2009 (see the electronic supplementary material S5). This comparison was feasible because of the relatively small number of fin whales taken by the Japanese scientific whaling program (13 from 2005/2006 to 2007/2008) and the large size of the Japanese market survey (80 products representing 19 individual fin whales). The comparison revealed an exact match at seven microsatellite loci and mtDNA haplotype, between the fin whale represented by the Seoul product and a fin whale represented by multiple products, first purchased in Japanese markets in September 2007 (see the electronic supplementary material S1, table S2). Based on the low probability of a match by chance calculated from the microsatellites of fin whales on the Japanese market (i.e. the probability of identity = 7 × 10^−11^, see the electronic supplementary material S1, table S4), we consider it highly probable that these products originated from the same individual whale.

## 4. Conclusions and proposal

The history of commercial whaling provides little assurance that international agreements will be honoured without an independent, transparent and robust system of monitoring. For a period of nearly 40 years, the Soviet Union defied its obligations to the IWC by falsifying catch records of more than 100 000 whales in the Southern Hemisphere alone (Clapham & Baker 2009). Japan's shore-based whaling stations falsified catch records for Bryde's and sperm whales into the 1980s (Kasuya 1999). Illegal, unreported or unregulated (IUU) exploitation continues under the cover of incidental fisheries bycatch and scientific whaling (Baker *et al*. 2000).

At its upcoming annual meeting in June 2010, the IWC is expected to consider a proposal to ‘set aside’ the existing categories of whaling for an interim period of 10 years, during which Japan, Norway and Iceland will continue to hunt under quotas that have yet to be determined (IWC 2010). DNA registers and market surveys are central elements in the management measures of this proposal. To date, however, there has been only a single independent effort to evaluate the functionality of the Norwegian DNA register (Palsbøll *et al*. 2006) and no reported effort to evaluate the functionality of the Japanese DNA registers. With this publication, we have submitted a request to the Government of Japan, through the IWC Secretariat, for access to the DNA registers (see the electronic supplementary material S3). In this, we also propose a third-party review of the DNA profiles by an independent laboratory with recognized expertise, such as the US Southwest Fisheries Science Center. A verified match of the DNA profiles from the fin, sei and Antarctic minke whale products to the Japanese DNA register would confirm an infraction of CITES regulations on trade of whale products. Alternatively, the absence of a match for one or more products would implicate an unknown source of IUU whaling, a situation requiring urgent investigation.
